# Elevated expression of MMP8 and MMP9 contributes to diabetic osteoarthritis progression in a rat model

**DOI:** 10.1186/s13018-021-02208-9

**Published:** 2021-01-19

**Authors:** Simin Luo, Wuji Li, Wenrui Wu, Qiping Shi

**Affiliations:** 1 Department of Bone and Joint Surgery, The First Affiliated Hospital, Jinan University, Guangzhou, 510632 China; 2 Department of Endocrine, The First Affiliated Hospital, Jinan University, Guangzhou, 510632 China

**Keywords:** Apoptosis, Diabetes, Osteoarthritis, Differentiation, MMP8, MMP9

## Abstract

**Background:**

Knowledge regarding the pathogenesis of osteoarthritis (OA) is very limited. Previous studies have shown that matrix metalloproteinase (MMP) 8 and MMP9 were upregulated in patients with diabetic OA. However, their regulatory functions and mechanisms in diabetic OA are not fully understood.

**Methods:**

Diabetic OA rats were constructed using a high-fat diet combined with streptozotocin (STZ) induction. Safranin O-Fast green staining was used to detect the pathological changes in rat knee cartilage. MMP8 and MMP9 overexpression vectors or siRNAs were injected into diabetic OA rats to overexpress or knockdown the expression of MMP8 and MMP9, which was verified by real-time quantitative PCR (RT-qPCR). The expression of MMP8 and MMP9, chondrocyte differentiation markers collagen type II alpha 1 (COL2A1) and collagen type I alpha 1(COL1A1), and antiapoptotic protein BCL2 were detected using immunohistochemistry (IHC), and the number of apoptotic cells was detected by the transferase-mediated d-UTP nick-end-labeling (TUNEL) assay.

**Results:**

High-fat diet combined with STZ-induced rats exhibited joint cartilage damage, morphological changes, and increased expression of MMP8 and MMP9. Overexpression of MMP8 and MMP9 in the joint cavity further aggravated the pathological morphological changes, decreased the expression of COL2A1 and COL1A1, increased the expression of BCL2, and promoted cell apoptosis in diabetic OA rats. The use of siRNA to inhibit MMP8 and MMP9 levels in the cartilage joints significantly reversed the decrease in COL2A1 and COL1A1 expression and partially reversed BCL2 expression and chondrocyte apoptosis.

**Conclusion:**

MMP8 and MMP9 promoted rat diabetic OA model. The underlying mechanism may be related to inhibiting cartilage differentiation and promoting chondrocyte apoptosis.

## Introduction

Osteoarthritis (OA) is the most common chronic degenerative disease in the elderly population [1]. Patients with OA are at an increased risk of the development of myocardial infarction [2]. In addition to affecting human physical health, OA also negatively affects mental health, such as causing suicidal ideation and perceived memory loss [3]. Although the harmful effects of OA have been recognized, the underlying mechanism of OA remains largely unknown, which may hinder the development of therapeutic drugs.

Many risk factors are known to be associated with OA development, including age, race, genetic, and diet. Recent evidence suggests that metabolic comorbidities, such as diabetes, are also significant risk factors for OA [4]. It has been shown that type 2 diabetes mellitus and OA frequently coexist [5], and type 2 diabetes mellitus is closely associated with accelerated knee cartilage matrix degradation, which is the main characteristic of OA [6, 7]. At the molecular level, it is currently believed that OA is associated with local and systemic low-grade inflammation [8]. It has been suggested that the production of matrix metalloproteinases (MMPs), which degrade cartilage matrix under inflammatory conditions, increases the degradation of the extracellular matrix. Studies suggested that Toll-like receptor 4 (TLR4) expression is elevated in osteoarthritic lesions of OA patients. And TLR4 signaling contributed to catabolic responses and resulted in elevated production of MMP [9]. It is widely accepted that the breakdown of the extracellular matrix by different MMPs is a characteristic feature of cartilage degeneration in OA that is closely related to the occurrence of OA [10–12]. However, whether MMPs promote diabetic OA remains unclear.

Among MMP members, MMP8 and MMP9 were found to be upregulated in the blood of patients with type 2 diabetes and metabolic syndrome [13, 14]. Moreover, they were also upregulated in the articular cartilage and synovial fluid in patients with OA [15–17], suggesting their possible involvement in diabetic OA. We hypothesize that MMP8 and MMP9 may play an important role in the pathogenesis of diabetic OA. Using lentiviral and siRNA methods, we explored the roles of MMP8 and MMP9 in a rat model of diabetic OA.

## Materials and methods

### Cell culture

The H9C2 cell line was purchased from the Cell Bank of the Shanghai Institute of Cell Biology, Chinese Academy of Sciences. Cells were maintained in Dulbecco’s modified Eagle’s medium in a 37 °C incubator containing 5% CO_2_.

### Plasmid construction and cell transfection

MMP8 and MMP9 overexpression plasmids constructed based on the LV003 plasmid were purchased from Guangzhou Yongnuo Company (Forevergen, Guangzhou, China). SiRNAs targeting MMP8 and MMP9 (MMP8, sense 5′-AAGCCATTGATGCAGCTGTTT-3′, antisense, 5′-AAACAGCTGCATCAATGGCTT-3′; MMP9, 5′-GCGAGACACTAAAGGCCAT-3′, antisense, 5′-ATGGCCTTTAGTGTCTCGC-3′) were synthesized from Genepharma (Shanghai, China). Cell transfection was performed using Lipofectamine 2000 (Thermo Fisher Scientific, WA, USA) according to the instructions. Twenty-four hours after transfection, qRT-PCR was performed to verify the overexpression and inhibition effect.

### Type 2 diabetes mellitus and diabetic OA animal model construction

An 8-week-old male Wistar rat was raised, and after 4 weeks of a high-fat diet, streptozotocin (STZ) 30 mg/kg (once a week for 2 weeks) was intraperitoneally injected. One week after the administration, nonfasting blood glucose was continuously monitored (blood glucose values greater than 16.7 mmol/L indicated successful generation of the type 2 diabetes mellitus animal model), and oral glucose tolerance tests were performed [18, 19].

### Animal experiment design

Twenty-four 8-week-old male Wistar rats were obtained from Beijing Vital River Laboratory Animal Technology Co., Ltd. Rats were randomly assigned to 4 groups, with 6 rats in each group. (1) Normal control group (NC): after 6 weeks of a normal diet, 20 μL of an empty lentiviral plasmid (3 μg/rat) were injected into the knee joint cavity every 7 days; (2) Diabetic OA group: 20 μL of an empty lentiviral plasmid (3 μg/rat) were injected into the knee joint cavity of diabetes rats every 7 days; (3) Diabetes-induced OA + MMP8/MMP9 overexpression group (diabetic OA + OE): 20 μL of MMP8 and MMP9 overexpression plasmids (3 μg/rat) were injected into the knee joint cavity of diabetes rats every 7 days. (4) Diabetes-induced OA + interfering MMP8/MMP9 group (diabetic OA + siRNA): 20 μL MMP8 and MMP9 siRNA (3 μg/rat) were injected into the knee joint cavity of diabetes rats once every 7 days. Animals in each group were sacrificed 4 weeks after plasmid or siRNA injection, and knee cartilage tissue and blood samples were collected. Cartilage tissue injury was evaluated using the Modified Osteoarthritis Research Society International (OARSI) scoring system by two independent observers.

### Fasting blood glucose and oral glucose tolerance test (OGTT)

The rats in each group were fasted for 24 h before the OGTT experiment. Fasting blood glucose was determined using a blood glucose meter (Elegance CT-X10, Germany, Convergent Technologies). For OGTTs, diH_2_O dissolved glucose (2 g/kg body weight; Sigma) and glucose were fed with a 20-gage stainless steel gavage feeding needle (Thermo Fisher Scientific). At 0, 15, 30, 60, and 120 min after glucose feeding (Elegance CT-X10, Convergent Technologies, Germany), OGTTs were measured using a blood glucose meter (Elegance CT-X10, Convergent Technologies, Germany).

### Safranin O-Fast green staining

The knee joints of rats were dissected mid-femur proximally and mid-tibia distally. Excess soft tissue was removed, and the knees fixed in 10% formalin at 4 °C overnight. The knees were then decalcified in a 0.45 M ethylenediamine tetra-acetic acid (EDTA) solution for three and a half weeks, changing solution daily. Following decalcification, samples were dehydrated and embedded in paraffin and then cut into sections. Samples were placed in the holder anterior side down, with the proximal end of the tibia flush with the bottom of the holder. Sections were taken every 6 μm, and sections from 150 to 550 μm were subsequently affixed to glass slides for staining. They were then stained with 0.5% Fast Green at room temperature for 20 min and then 0.5% Safranin O for 5 min in order to visualize the articular surface and surrounding structures. The morphological changes in cartilage tissues were observed under a microscope (CCD TP510, Chongqing OPTEC Co, Ltd, China).

### Real-time quantitative PCR (RT-qPCR)

Total RNA was isolated using Trizol (Invitrogen, Carlsbad, CA, USA) and reverse transcribed into cDNA using a QuantiTect Reverse Transcription Kit (Qiagen, Valencia, CA, USA). The resultant cDNAs were amplified using a miScript SYBR Green PCR Kit (Qiagen) according to the manufactures’ instructions. MMP8 and MMP9 were amplified using specific primers that are listed in Table 1. GAPDH was used as an internal control. The relative expression levels of MMP8 and MMP9 were calculated using the 2^−ΔΔCt^ method.

**Table 1:** Specific primers

Gene ID	primers	Sequence (5′-3′)	Length (bp)
MMP-8	Rat-MMP-8-F	CCATGGATCCAGGTTACCCCACT	112
	Rat-MMP-8-R	TGTGGTCCACTGAAGAAGAGGAAGA	
MMP-9	Rat-MMP-9-F	GGATGTTTTTGATGCCATTGCTG	127
	Rat-MMP-9-R	CCACGTGCGGGCAATAAGAAAG	
GAPDH	Rat-GAPDH-F	GCAAGAGAGAGGCCCTCAG	74
	Rat-GAPDH-R	TGTGAGGGAGATGCTCAGTG	

### Immunohistochemistry (IHC) assay

The method of sampling is the same as Safranin O-Fast green staining. Articular cartilage tissues were fixed in 4% paraformaldehyde and embedded in paraffin. The block was cut into 6 μm sections, and slides were prepared. Slides were incubated in primary antibodies for MMP8 (BA2201, dilution 1:100, Boster), MMP9 (ab38898, dilution 1:100, Abcam), COL1A1 (ab23446, dilution 1:100, Abcam), COL2A1 (ab34712, dilution 1:100, Abcam), and BCL2 (bs-0032R, dilution 1:200, Bioss) at 4 °C overnight. The next day, slides were washed three times and incubated with polyclonal antibodies against rabbit IgG-HRP (#SV0002, Boster) and mouse IgG-HRP (#SV0001, Boster) at room temperature for 1 h. Slides were then stained with 1 μM DAPI (Thermo Fisher Scientific, Waltham, MA, USA) to visualize cell nuclei.

### Transferase-mediated d-UTP nick-end-labeling (TUNEL) assay

The apoptotic level in the bone tissue was assessed using the TUNEL assay. Tissues from articular cartilage were embedded in paraffin, dewaxed, and hydrated. Apoptotic cells were stained using a TdT Frag DNA Fragmentation Imaging kit (Sigma Aldrich) according to the manufacturers’ instructions. The proportion of positive cells in five random fields at high magnification from 3 representative slides was calculated.

### Statistical analysis

All data were expressed as means ± SE and analyzed with Graph Pad Prism (version 7.0). Differences between two groups were analyzed using Student’s *t* test. For comparisons among more than three groups, data were analyzed using one-way ANOVA followed by Tukey’s post hoc test. *p* < 0.05 was considered to indicate statistical significance.

## Results

### Upregulated expression of MMP8/MMP9 in the knee cartilage of diabetic OA rats

**Fig. 1:**
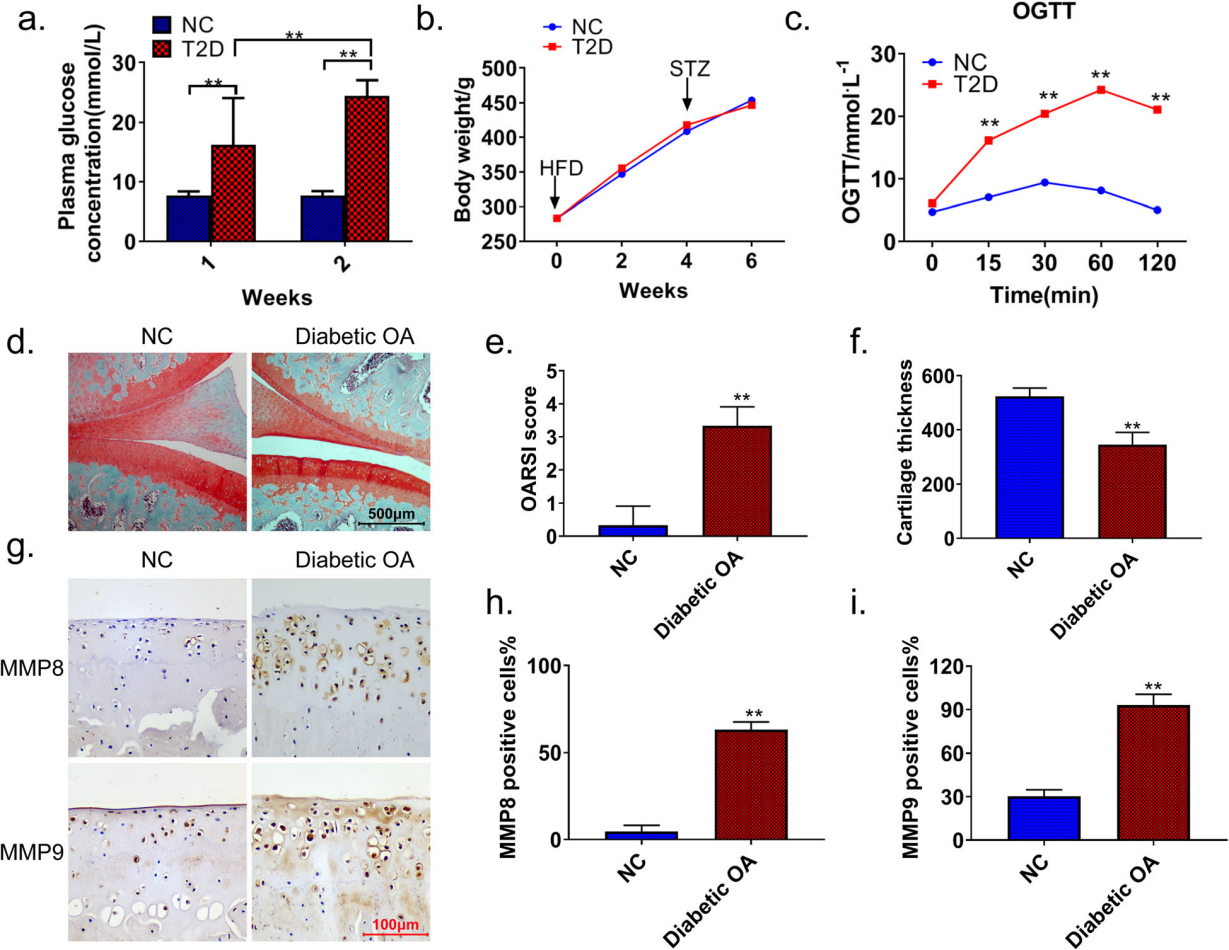
Upregulated expression of MMP8 and MMP9 in the knee joint cartilage of diabetic OA rats. **a** After 4 weeks of a high-fat diet, blood glucose values of the model (STZ) and control (NC) rats were monitored at 1 and 2 weeks after modeling. **b** The body weights of rats in each group were recorded after 1–6 weeks of OA induction. **c** OGTT results of STZ and NC rats. **d** Safranin O-Fast green staining was used to detect articular cartilage lesions in diabetic OA and NC rats. OARSI scores (**e**) and articular cartilage thickness (**f**) in diabetic OA and NC rats. **g**–**i** IHC showing the expression levels of MMP8 and MMP9 in the joint tissues of diabetic OA and NC rats. Image-Pro Plus was used to quantify the expression. Diabetic OA diabetic osteoarthritis, T2D high-fat diet combined with STZ, NC normal control, OGTT oral glucose tolerance test, MMP matrix metalloproteinase. **p < 0.01

A high-fat diet combined with STZ was used to induce diabetic OA in rats. After 4 weeks of a high-fat diet, STZ injection was given for 2 consecutive weeks. Within 2 weeks after the injection, fasting blood glucose levels in rats induced by a high-fat diet combined with STZ (T2D) were significantly higher than those in NC rats (*p* < 0.01) (Fig. 1a). As shown in Fig. 1b, the 6-week weight change was not significantly different between the T2D and NC groups. In contrast, the OGTT results were significantly different between the two groups (*p* < 0.01) (Fig. 1c). Safranin O-Fast green staining showed that the knee cartilage structure in the normal group (NC) was intact, and the matrix composition was evenly distributed. However, in the diabetic OA rats, we observed weakened and unevenly distributed matrix staining, increased chondrocyte clusters, and disordered arrangement of chondrocytes. The OARSI score showed that the diabetic OA group had knee injuries and decreased cartilage thickness (Fig. 1d–f). Of note, the IHC results showed that the number of MMP8- and MMP9-positive cells was significantly increased in the knee joint cartilage of the diabetic OA rats (*p* < 0.01) (Fig. 1g–i).

### High expression of MMP8/MMP9 aggravates knee cartilage injury in diabetic OA rats

**Fig. 2:**
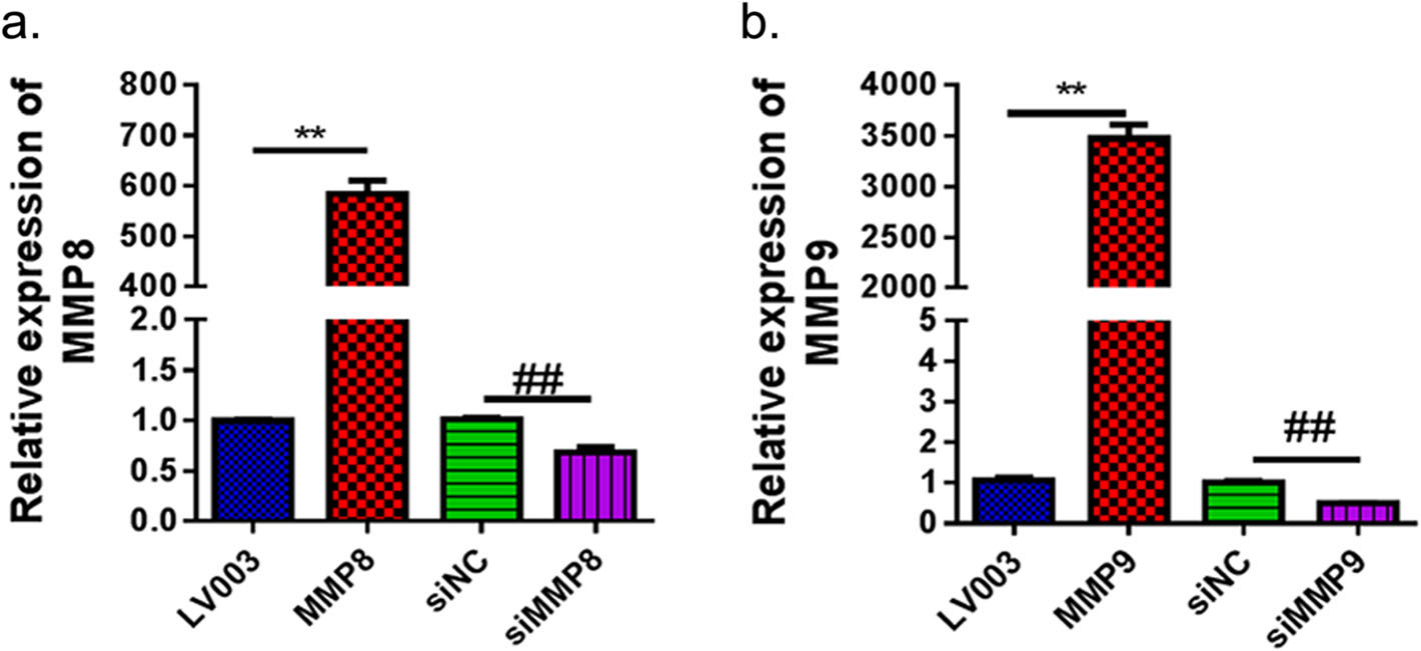
qRT-PCR verified the overexpression and inhibition of MMP8 (**a**) and MMP9 (**b**) expression in H9C2 cells. LV003, lentivirus-based control empty vector; MMP8/MMP9, MMP8/MMP9 overexpression vector constructed based on the LV003 vector; siNC, scramble siRNA; siMMP8/siMMP9, cells transfected with MMP8/MMP9 siRNA fragments. MMP matrix metalloproteinase. **p < 0.01, ^##^*p* < 0.01

To observe the effect of MMP8/MMP9 overexpression on the knee cartilage in diabetic OA rats, we generated MMP8/MMP9 overexpression plasmids and synthesized MMP8 and MMP9 siRNAs. As shown in Fig. 2a, the constructed MMP8 overexpression plasmid and MMP8 siRNA significantly increased and decreased the expression of MMP8 mRNA, respectively, in H9C2 cells (*p* < 0.01). Similar results were observed in H9C2 cells transfected with the MMP9 overexpression plasmid and MMP9 siRNA (Fig. 2b).

**Fig. 3:**
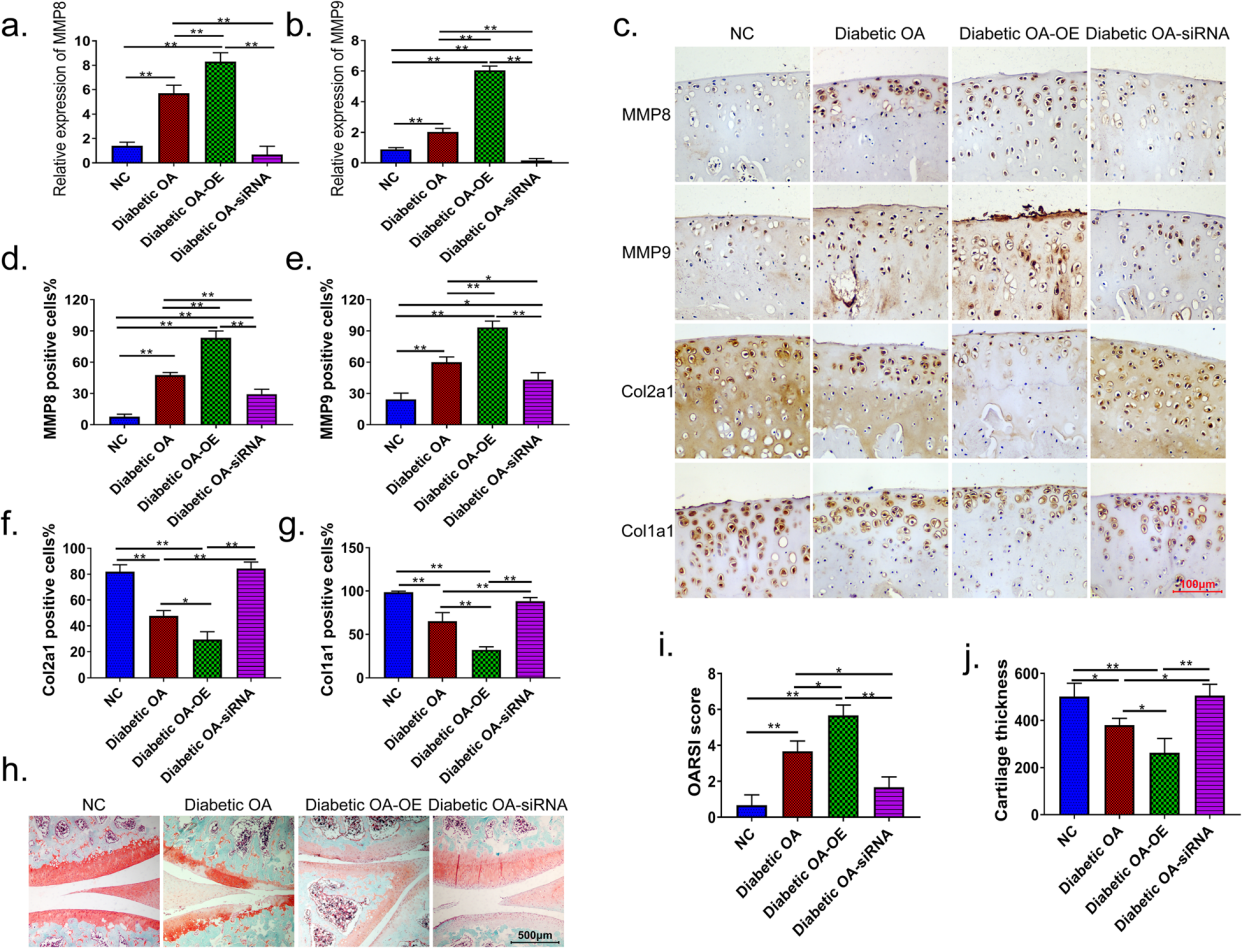
Effects of MMP8 and MMP9 on the protein expression of cartilage markers and morphology of the knee joints in diabetes-induced OA rats. qRT-PCR was used to detect the expression levels of MMP8 (**a**) and MMP9 (**b**) in the diabetic knee joint cavity. **c** IHC showing the effect of overexpression plasmids and siRNAs of MMP8 and MMP9 on the expression of MMP8, MMP9, COL2A1, and COL2A1 in the knee joint cavity. **d**–**g** Quantitative analysis of IHC micrographs using Image-Pro Plus software. **h** Safranin O-Fast green staining was used to detect changes in the knee cartilage structure. i–j Quantitatively analysis of Safranin O-Fast green staining using Image-Pro Plus software. Diabetic OA diabetic osteoarthritis, *NC* normal control, Diabetic OA-OE diabetic OA and the MMP8 and MMP9 overexpressing group, Diabetic OA-siRNA diabetic OA interferes with the expression of MMP8/MMP9, COL2A1 collagen type II alpha 1, COL1A1 collagen type I alpha 1, MMP matrix metalloproteinase. *p < 0.05; **p < 0.01

We then injected MMP8 and MMP9 overexpression plasmids or siRNAs into the knee joint cavity of rats to determine the effects of these treatments on the knee joint cartilage in diabetic OA rats. The expression of MMP8 and MMP9 were significantly increased in diabetic OA rats compared with that in NC rats (*p* < 0.01). In articular cartilage tissue, overexpression plasmids or siRNAs of MMP8 and MMP9 significantly increased or decreased the expression levels of MMP8 and MMP9, respectively (*p* < 0.01) (Fig. 3a, b). IHC results revealed that the expression of COL2A1 and COL1A1 was decreased, whereas MMP8 and MMP9 levels were increased in diabetic OA rats. The overexpression of MMP8 and MMP9 further inhibited the expression of cartilage markers COL2A1 and COL1A1 in diabetic OA rats, whereas MMP8 and MMP9 siRNAs restored normal COL2A1 and COL1A1 expression in model rats (Fig. 3c–g). Safranin O-Fast green staining showed that the cartilage in the NC knees was intact, and the matrix components were evenly distributed. However, in the diabetic OA and the MMP8 and MMP9 overexpressing group (Diabetic OA-OE), matrix staining was weakened and unevenly distributed. In addition, the superficial surface of the joint was lost, chondrocyte clusters were increased, and chondrocytes were disorderly arranged. In contrast, the thicknesses of cartilage and chondrocyte in diabetic OA rats with reduced expression of MMP8/MMP9 (Diabetic OA-siRNA) were increased (Fig. 3h–j).

### MMP8/MMP9 promotes the apoptosis of chondrocytes in the knee joint of diabetic OA

**Fig. 4:**
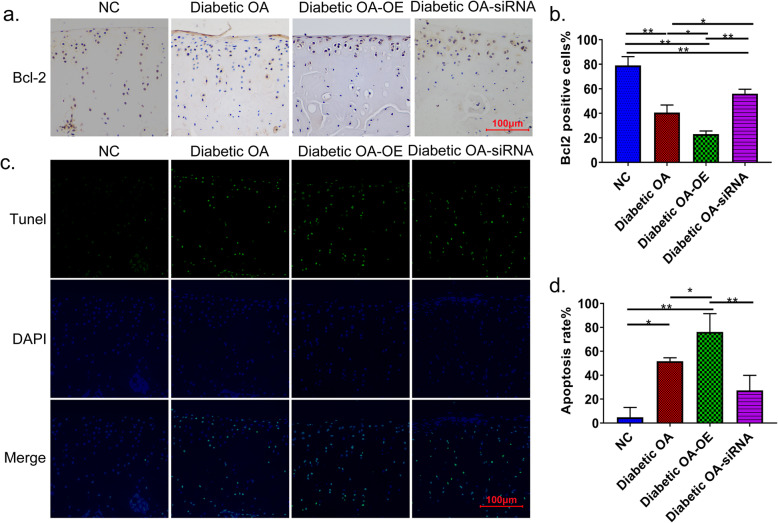
The effect of MMP8 and MMP9 on the apoptosis of chondrocytes in the knee joint of diabetes-induced OA rats. **a** IHC showing the effects of MMP8/MMP9 overexpression and inhibition on the expression of BCL2 in knee joint cartilage. **b** Image-Pro Plus software was used for the quantitative analysis of BCL2. **c** TUNEL assay results showing the effect of overexpression and inhibition of MMP8 and MMP9 on the number of apoptotic cells. **d** Quantitative analysis of the number of apoptotic cells. *p < 0.05; **p < 0.01

To investigate whether MMP8 and MMP9 are related to apoptosis in diabetic OA in rats, we used IHC to detect the expression of antiapoptosis-related proteins and TUNEL to detect the number of apoptotic rat knee chondrocytes. As shown in Fig. 4a, b, the antiapoptotic protein BCL2 was significantly decreased in the knee joint cartilage of diabetic OA rats (*p* < 0.01). Moreover, the expression was lowest in diabetic OA rats overexpressing MMP8 and MMP9 (*p* < 0.01). And inhibiting the expression of both MMP8 and MMP9 partially restored the expression level of BCL2.

The TUNEL assay results further confirmed that the overexpression of MMP8 and MMP9 significantly aggravated the apoptosis of chondrocytes in the knee joint of diabetic OA rats (*p* < 0.01), whereas inhibiting the expression of MMP8 and MMP9 showed a protective effect on chondrocyte apoptosis in diabetic OA rats (Fig. 4c, d).

## Discussion

In this study, we found that MMP8 and MMP9 were upregulated in the knee joints of diabetic OA rats. The overexpression of MMP8 and MMP9 aggravated cartilage injury in the knees of diabetic OA rats. Specifically, we observed downregulation of the expression of cartilage markers COL2A1 and COL1A1 and the promotion of pathological changes in knee structure and morphology. In contrast, interfering with the expression of MMP8 and MMP9 alleviated the cartilage damage in diabetic OA rats. Furthermore, we found that the overexpression of MMP8 and MMP9 inhibited the expression of antiapoptotic protein BCL2 and increased the number of apoptotic chondrocytes in diabetic OA rats, whereas inhibition of MMP8 and MMP9 partially reversed this proapoptotic effect.

The mechanism of diabetic OA has not yet been fully elucidated. At present, accelerated knee cartilage degradation is the main feature of patients with type 2 diabetes mellitus [6]. In particular, MMPs are considered to play an important role in the process of cartilage decomposition during the induction of degenerative cartilage changes that occur in OA [20]. It is believed that this is because cartilage extracellular matrix is mainly composed of proteoglycans, including collagen, and MMPs are the main proteolytic enzyme of these proteins that can degrade type II fibrillar collagen, IX, XI, and VI collagen, and other secondary collagens [21]. It has been found that MMP1 and MMP13 promote the occurrence and development of OA by degrading cartilage extracellular matrix [22–24]. However, there are still many MMP family members whose functions are not fully understood. By overexpressing and suppressing MMP8 and MMP9 levels in diabetic OA rats using plasmid and siRNA transfections, we found that MMP8 and MMP9 aggravated the cartilage joint damage in diabetic OA, whereas inhibiting MMP8 and MMP9 expression showed a protective effect against the articular cartilage. Our research provides new evidence that MMP family members are involved in regulating diabetic OA. In addition, we found that MMP8 and MMP9 were upregulated in the cartilage tissue of the diabetic OA rats. This finding expanded the existing understanding and provided evidence that in addition to their expression in OA and diabetic patients, MMP8 and MMP9 are also overexpressed in diabetic OA rats.

In the present study, we observed that MMP8 and MMP9 aggravated the pathological changes in cartilage morphology and the decrease in cartilage thickness in diabetic rats. It is currently believed that in mature articular cartilage, chondrocytes play an important role in maintaining the cartilage-specific matrix phenotype [25]. Therefore, stabilizing the number of chondrocytes is particularly important for maintaining the chondrocyte phenotype. We examined the effect of MMP8 and MMP9 expression intervention on the number of diabetic OA knee chondrocytes. Since COL2A1 and COL1A1 are both markers of chondrocytes [26, 27], we measured the expression of COL2A1 and COL1A1 to reflect the number of chondrocytes in the knee. Our results show that overexpression of MM8 and MMP9 inhibits the expression of the diabetic OA cartilage markers COL2A1 and COL1A1, suggesting the inhibition of cartilage differentiation. Furthermore, inhibiting the expression of MMP8 and MMP9 can partially reverse the decrease in COL2A1 and COL1A1 expression. Our results suggest that the reduced number of chondrocytes may contribute to the morphological and pathological changes and the reduction in cartilage thickness.

The MMP family contains multiple members with diverse functions that mainly include the regulation of cell behaviors, such as cell proliferation, migration (adhesion/dispersion), differentiation, apoptosis, and host defense [12, 25]. MMP8 is a type 2 collagenase. In previous studies, it was found that the expression level of MMP8 in obese patients is increased and that it can promote insulin resistance by cleaving insulin receptors [28]. Although mutations in MMP8 are known to be associated with OA, the current understanding of MMP8 in OA and diabetic OA and its impact on chondrocyte damage is still relatively limited. MMP9 is a type IX collagenase. In diabetic mice, the increased activity of MMP9 has been shown to play a role in promoting endothelial cell apoptosis and endothelial dysfunction [29]. In OA, MMP9 was also found to be significantly upregulated [30]. However, similar to MMP8, the specific functions of MMP9 in OA remain poorly understood. Notably, some studies suggest that MMP8 and MMP9 have a synergistic effect, and the upregulation of MMP9 expression is observed in MMP8-deficient mice [31]. Considering this effect, we simultaneously overexpressed and inhibited the expression of MMP8 and MMP9. We found that the expression of antiapoptotic protein BCL2 was suppressed, and the apoptosis of diabetic OA rat chondrocytes was promoted by MMP8 and MMP9 overexpression and alleviated by siRNA treatments. Our findings suggest that MMP8 and MMP9 exhibit a proapoptotic effect on diabetic OA.

However, this study has some limitations. Based on the methods and results we have got, we evaluated OA severity at single time point, so this paper should be described as a pilot study. But these results might provide some assistance for further study on the mechanism of MMP8 and MMP9 in diabetic osteoarthritis. Therefore, we will make further experiments, such as longitudinal evaluation of OA severity, rather than at single time point, to study the mechanism of MMP8 and MMP9 in diabetic osteoarthritis and the comprehensive experiments will be obtained and reported in future.

## Conclusion

In conclusion, our study found that MMP8 and MMP9 are upregulated in the knee cartilage of diabetes-induced OA rats. MMP8 and MMP9 aggravate the injury of the knee joint cartilage and promote the apoptosis of articular chondrocytes in diabetes-induced OA rats. The underlying mechanism of the effects of MM8 and MMP9 on diabetic OA articular cartilage may be related to the inhibition of cartilage differentiation and enhanced chondrocyte apoptosis. However, the detailed mechanisms require further study.

## Data Availability

The datasets generated during and/or analyzed during the current study are available from the corresponding author on reasonable request.
